# The CD28 Transmembrane Domain Contains an Essential Dimerization Motif

**DOI:** 10.3389/fimmu.2020.01519

**Published:** 2020-07-16

**Authors:** Scott A. Leddon, Margaret M. Fettis, Kristin Abramo, Ryan Kelly, David Oleksyn, Jim Miller

**Affiliations:** Department of Microbiology and Immunology, David H. Smith Center for Vaccine Biology and Immunology, University of Rochester Medical Center, Rochester, NY, United States

**Keywords:** CD28, transmembrane domain, protein dimerization, FRET, CRISPR, knock-in mice

## Abstract

CD28 plays a critical role in regulating immune responses both by enhancing effector T cell activation and differentiation and controlling the development and function of regulatory T cells. CD28 is expressed at the cell surface as a disulfide linked homodimer that is thought to bind ligand monovalently. How ligand binding triggers CD28 to induce intracellular signaling as well as the proximal signaling pathways that are induced are not well-understood. In addition, recent data suggest inside-out signaling initiated by the T cell antigen receptor can enhance CD28 ligand binding, possibly by inducing a rearrangement of the CD28 dimer interface to allow for bivalent binding. To understand how possible conformational changes during ligand-induced receptor triggering and inside-out signaling are mediated, we examined the CD28 transmembrane domain. We identified an evolutionarily conserved YxxxxT motif that is shared with CTLA-4 and resembles the transmembrane dimerization motif within CD3ζ. We show that the CD28 transmembrane domain can drive protein dimerization in a bacterial expression system at levels equivalent to the well-known glycophorin A transmembrane dimerization motif. In addition, ectopic expression of the CD28 transmembrane domain into monomeric human CD25 can drive dimerization in murine T cells as detected by an increase in FRET by flow cytometry. Mutation of the polar YxxxxT motif to hydrophobic leucine residues (Y145L/T150L) attenuated CD28 transmembrane mediated dimerization in both the bacterial and mammalian assays. Introduction of the Y145L/T150L mutation of the CD28 transmembrane dimerization motif into the endogenous CD28 locus by CRISPR resulted in a dramatic loss in CD28 cell surface expression. These data suggest that under physiological conditions the YxxxxT dimerization motif within the CD28 transmembrane domain plays a critical role in the assembly and/or expression of stable CD28 dimers at the cell surface.

## Introduction

The functional importance of CD28 in controlling the set point for immune responsiveness has been well-established. T cell encounter with peptide-MHC ligands in the absence of an ongoing innate immune response does not lead to effective T cell activation and rather favors the induction of tolerance. CD28 has been shown to have a wide range of important functional consequences on T cell activation, survival, metabolic activity, differentiation, and effector function (1–7). CD28 is also required for the thymic maturation of natural killer T cells (8, 9), the generation of T follicular helper cells, T follicular regulatory cells, and the dynamic regulation of the germinal center (10–14), and for the survival of long lived plasma cells in the bone marrow (15–17). Although CD28 was initially identified as a positive regulator of effector T cell responses, it is now known that CD28 is also a critical factor in T regulatory cell (Treg) development, peripheral survival, and function (18–24). However, despite considerable interest and effort, the mechanisms of CD28 signaling are not well-understood (25, 26). Likewise, it is not clear how ligand binding initiates CD28 signal transduction. Although CD28 is a disulfide linked homodimer, a soluble, recombinant form of CD28 can only interact with ligand monovalently (27). Based on the crystal structure of the soluble form of CD28 (28), it is thought that the length and rigidity of the ligands for CD28 (CD80 and CD86) and the angle in which these ligands bind results in steric interference at the distal end of CD80 and CD86. Consistent with this model, it was found that CD28 can bind truncated CD86 bivalently, indicating that both ligand binding sites in the CD28 dimer are functional (27).

Two models have been proposed for ligand-induced receptor triggering and the induction of downstream signaling from monovalent ligand-binding receptors. In some cases, ligand binding can induce the formation of homotypic or heterotypic dimers or multimeric complexes, either directly, because the ligand itself is multimeric, or indirectly by inducing a conformational change in the receptor that reveals an intrinsic interaction motif (29–33). Alternatively, monovalent receptors may exist in a preformed dimer and ligand binding induces a conformational change that is transduced through the transmembrane (TM) domain (34, 35). The two CD28 ligands differ in their structure and affinity for CD28. CD80 has a higher affinity for CD28 and has a tendency to form non-covalent dimers, both within the crystal structure (36) and when expressed at the cell surface (37). In contrast, CD86 both crystalizes and is primarily expressed at the cell surface as a monomer (37, 38). Thus, interaction with dimeric CD80 has the potential to cross-link two monovalent CD28 dimers, whereas interaction with CD86 cannot. However, both CD80 and CD86 have been shown to be able to induce CD28 signaling (39–41) and some lines of evidence suggest that CD28 preferentially interacts with the monomeric ligand, CD86 (42). Based on the monovalent binding model for CD28, these data would predict that ligand binding itself, rather than receptor cross-linking, is sufficient to induce CD28 triggering.

An alternative model for CD28 ligand binding is that in the context of plasma membrane expression, T cell receptor (TCR) signaling may induce a conformational change in CD28 that allows for bivalent binding (43, 44). The ability of signaling from one receptor to enhance ligand binding of an independent receptor has been well-documented for integrins and is referred to as inside-out signaling (45, 46). During integrin activation, signals from TCR or from chemokine receptors initiate a reorientation of the cytosolic domains of the integrin heterodimer, that is then transduced through the TM domains and results in a structural change in the extracellular domains (45, 46). We have shown that TCR signaling can also induce a change in the orientation of the cytosolic domains within the CD28 dimer and can enhance CD28 ligand binding (43, 44). However, unlike integrins that contain multiple extracellular domains that can undergo structural rearrangements, each CD28 monomer contains only a single immunoglobulin-like domain (28), that is unlikely to undergo large internal structural changes. Recent data from our lab indicate that during T cell activation, CD28 may undergo a reorientation of the extracellular dimer interface, allowing for bivalent ligand binding (43). Consistent with this model, CD28 dimers that contain only a single functional ligand binding site are poorly recruited to the immunological synapse (43). In the context of cell-cell interactions, this change from monovalent to bivalent binding can result in a >100-fold increase in effective receptor-ligand binding (47, 48). Thus, TCR-induced increase in valency could result in an increase in the avidity of CD28 ligand binding and could account for enhanced CD28 ligand binding, cross-linking, triggering, and initiation of downstream signaling.

In either model of CD28 ligand binding and triggering, understanding the nature and regulation of the dimer interaction sites is paramount to understanding the potential structural changes that might mediate inside-out and/or outside-in signaling. CD28 has two known dimer interaction sites. The CD28 homodimer is covalently linked by a single disulfide bond within the membrane proximal stalk region. However, it has been reported that mutation of this disulfide bond does not interfere with stable dimer formation (49). In addition, within the extracellular domain there is a hydrophobic dimer interface that was resolved in the CD28 crystal structure (28).

Recent data have demonstrated that, in addition to their membrane localization and spanning functions, TM domains can play important functional roles both in multimeric protein assembly and in signal transduction (50–52). For example, a GxxG motif first identified in the TM of Glycophorin A (GpA) (53–55) facilitates dimerization of a number of cell surface proteins, including CD4 (56) and MHC class II (57, 58). Ionic TM interaction motifs have been in identified in TCR/CD3 complex (59–61) and polar interactions in MHC class II-associated invariant chain (62, 63) and DAP12 (64). TM domain interactions can also be regulated dynamically during cellular activation, inducing changes to ligand binding in integrins (65–68) and transducing proximal signaling events through the TCR/CD3 complex (69) and other cell surface receptors (70–75). Interestingly, evolutionary analysis of CD28 sequences revealed several positively selected sites that mapped to the CD28 TM, suggesting a function role for the CD28 TM domain (76).

In this report, we show that the CD28 TM domain contains a highly conserved dimerization motif that is shared with CTLA-4 and structurally related to the CD3ζ TM dimerization motif. Furthermore, mutation of this TM dimerization in the endogenous CD28 locus results in a dramatic loss in steady state CD28 expression at the cell surface suggesting that the TM domain is critical for the assembly and/or steady state expression of stable CD28 dimers.

## Materials and Methods

### ToxLuc Assay

The ToxLuc system and the constructs containing the wild type (WT) GpA (LIIFGVMAGVIGT), mutant GpA (G83I; LIIFGVMAIVIGT) and polyalanine (A17; 17 alanines) TM domains were generously provided by Samuel Campos (U Arizona) (77). WT (FWALVVVAGVLFCYGLLVTVALCVIWT) and Y145L/T150L (YT/LL) mutated (FWALVVVAGVLFCLGLLVLVALCVIWT) mouse CD28 TM domains and WT (VLVVVGGVLACYSLLVTVAFIIF) and YT/LL mutated (VLVVVGGVLACLSLLVLVAFIIF) human CD28 TM domains were cloned into the ToxLuc vector and confirmed by DNA sequencing. Bacterial lysates were generated in NT326 cells and assayed for fusion protein expression by western blot detected with a maltose binding protein (MBP)-specific rabbit antibody (New England Biolabs, Beverly, MA). Lysates were assayed for luciferase expression by ONE-Glo luciferase (Promega, Madisen, WI).

### Retroviral Transduction and FRET Assay

A full-length cDNA clone for human CD25 (hCD25, generously provided by Warren Leonard, NIH) was fused at the 3′ end to monomeric YFP or CER via a 7 amino acid linker (AGPGSTG) or to both CER and YFP that were separated by a 5 amino acid linker (GGGGG). To generate hCD25 chimeras containing the CD28 or CTLA-4 TM domains, the hCD25 TM domain (QVAVAGCVFLLISVLLLSGLTW) was replaced with either the WT (WALVVVAGVLFCYGLLVTVALCVIW) or YT/LL (WALVVVAGVLFCLGLLVLVALCVIW) CD28 TM domain or WT (FLLWILVAVSLGLFFYSFLVTAVSLS) or YT/LL (FLLWILVAVSLGLFFLSFLVLAVSLS) CTLA-4 TM domains by overlapping PCR. Likewise, the YT/LL, C123S, and double (YT/LL/C123S) mutations were introduced into WT mCD28 by overlapping PCR and fused at the C-terminus to CER and monomeric YFP with a 4 amino acid linker (RSTG). All DNA constructs were confirmed by DNA sequencing. To confirm deletion of the disulfide bond in the C123S mutation, both WT and C123S mouse CD28 were fused to an HA tag at the C-terminus and NP40 cell lysates were analyzed on a western blot probed with a rabbit anti-HA mAb (Cell Signaling, Danvers, MA).

The sequences encoding the fusion proteins were cloned into the MSCV retroviral vector, MIGR1 (78), while deleting the virally encoded IRES-GFP. Retroviruses were prepared by transient transfection into Phoenix cells and concentrated by PEG precipitation (Retro-X, Clontech, Mountain View, CA). A CD28-negative DO11.10 T cell hybridoma cell line was stably transduced with the recombinant retroviruses by centrifugation at 2,000 rpm (850 g) for 60 min in the presence of 4 μg/ml polybrene. Levels of hCD25 or mouse CD28 expression on the cell surface was determined by flow cytometry after staining with either anti-human CD25-PE-Cy5 (clone M-A25, BD Bioscience, San Jose, CA) or with anti-mouse CD28-APC (clone 37.51, Tonbo Biosciences, San Diego, CA). For FRET experiments hybridomas were first transduced with CER expressing constructs and sorted for similar levels of CER by flow cytometry. These cells were used as CER alone controls or were re-transduced with YFP constructs to generate dual YFP/CER expressing lines. The parent hybridoma was also transduced with YFP constructs to generate YFP alone controls or with the hCD25-CER-YFP linked construct to generate a positive control for FRET. Cells were maintained in high glucose DMEM (Thermo Fisher Scientific, Waltham, MA) supplemented with 10% FBS (Hi-clone, Marlborough, MA), 50 μM 2-ME, 22 mM HEPES, 2 mM glutamine, and 0.1 mM non-essential amino acids (Corning Life Sciences, Tewksbury, MA).

FRET was analyzed on a 12-color LSRII flow cytometer (BD Biosciences). CER signals were detected with 450/50 BP emission filter after samples were excited with 405 nm laser. YFP signals were detected with an inline 505 nm long pass and a 525/50 nm emission filter after excitation with a 488 nm laser. FRET signals were detected with an inline 505 nm long pass and a 550/50 nm emission filter after excitation with a 405 nm laser. Data was analyzed using either FlowJo 8.6 or 10.2 software (Tree Star, Inc., Ashland, OR). Relative FRET efficiency (eFRET) was represented by FRET signal/FRET signal + CER signal.

CD4-positive cells were purified by negative selection (MACS, Miltenyi, Bergisch Gladbach, Germany) from CD28-deficient, DO11.10 TCR transgenic or CD28-deficient OTII TCR transgenic mice and stimulated for 2 days with antigen presented by irradiated syngeneic spleen cells and 10 U/ml rhIL-2. Live cells were isolated on a ficoll gradient and transduced with retroviruses as described above. Retroviruses contained either WT or YT/LL TM domains, the C123S mutation, or both the YTLL and C123S mutations in either full-length mouse or human CD28. The IRES-GFP cassette was included in these retroviruses to control for the efficiency of transduction and relative expression of retrovirally encoded mRNA. T cells were harvested 7–10 days after initial activation, stained with anti-mouse-Alexa647 or anti-human CD28-PE-Cy5 (BD Biosciences) and analyzed by flow cytometry to assess relative levels of CD28 surface expression and GFP.

### YT/LL Knock-In Mice

The YT/LL mutation was knocked into the endogenous CD28 locus in inbred C57BL/6 mice by CRISPR. The sgRNA [5′-TTATGGCTTGCTAGTGACAG(TGG)-3′], single stranded donor oligonucleotide with Y155, T160, and endogenous PAM sites mutated (5′-ACTCAGTCATCTCCTAAGCTGTTTTGGGCACTGGTCGTGGTTGCTGGAGTCCTGTTTTGTctaGGCcTGCTAGTGctgGTaGCTCTTTGTGTTATCTGGGTAAGAGGAGCAACATTGCTTTTATGTAACTTCTCTGCG-3′) and purified CAS9 were microinjected into fertilized eggs. Offspring were screened by PCR and introduction of the YT/LL mutation was confirmed by DNA sequencing. Three independent founder lines were generated and backcrossed to WT C57BL/6 mice.

Single cell suspensions were prepared from spleen and thymus and treated with ACK lysis buffer (0.15 M NH_4_Cl/1 mM KHCO_3_/0.1 mM Na_2_-EDTA in H_2_O, pH 7.2) for 5 min to deplete red blood cells. Except where noted all staining, washes, and resuspensions were performed in FACS buffer (1X PBS with 2% FBS). Cells were pre-stained with Ghost Dye Violet 510 (Tonbo Biosciences, San Diego, CA) to exclude dead cells and with anti-CD16/CD32 (2.4G2, Tonbo) to block FcR binding. Cells were then stained with a cocktail of CD4-PE-cy7 (clone RM4-5, Tonbo), CD8α-biotin (clone 53-6.7, Tonbo), CD44-APC-Cy7 (clone IM7, Tonbo), CD25-PE (clone PC61, BD bioscience), and CD28-BV421 (clone 37.51, BD), followed by streptavidin-APC (BD), and then fixed and permeabilized with Transcription Factor Buffer Set (BD) and stained with FOXP3- Alexa488 (clone MF23, BD). Cells were run on an LSRII flow cytometer and data were analyzed using either FlowJo 8.6 or 10.2 software.

For real-time PCR analysis, RNA was prepared from spleen and thymus isolated from two independent WT, CD28YT/LL knock-in, and CD28 knockout mice by TRIzol (Invitrogen, Carlsbad, CA) extraction. The quality of the RNA was evaluated on formaldehyde denatured agarose gels, cDNA was prepared using the high capacity cDNA reverse transcription kit from Applied Biosystems (Foster City, CA), and the level of CD28 mRNA was quantified using TaqMan primers (Applied Biosystems) that span either exons 1-2 or exons 3-4 on a CFX Connect Real Time System (Biorad, Hercules, CA). The level of CD28 mRNA was normalized to GAPDH within each sample and then normalized to the mean of the two WT samples.

## Results

**Figure 1:**
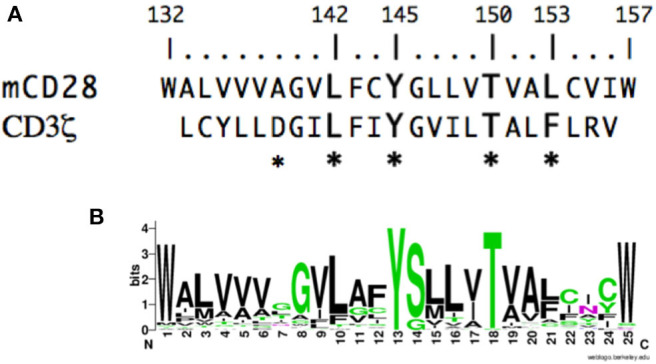
The CD28 TM domain contains a highly conserved motif that shares homology with the CD3ζ dimerization motif. **(A)** The amino acid sequence of the mouse CD28 TM segment is shown on the top and the sequence of the human CD3ζ TM is shown below. The numbering corresponds to the mature mouse CD28 protein and the asterisks identify residues within CD3ζ that are involved in dimerization. **(B)** A WebLogo consensus sequence of CD28 TM sequences from 51 diverse species ranging from human to coelacanths (see Supplementary Table 1 for a complete listing of sequences included). The WebLogo was created at http://weblogo.berkeley.edu; black, hydrophobic (AVLIPWFM); green, polar (GSTYCQN); red, acidic (DE); and blue, basic (KRH) side chains.

To gain insight into the molecular events that might mediate inside-out or outside-in signaling in CD28, we analyzed the potential contribution of the CD28 TM domain. If the CD28 TM contains a functional motif, it might be expected to be evolutionarily conserved. To test this possibility, we compared the sequence of the CD28 TM across species to search for a potentially conserved functional motif. CD28 homologs can be identified in many species down to primitive vertebrates and the predicted hydrophobic TM segment is readily identified with highly conserved N- and C-terminal tryptophan residues (Figure 1A). Tryptophan and other aromatic amino acids are commonly found at the boundaries of TM domains (79, 80) and the predicted CD28 TM domain of 25 amino acids is typical of plasma membrane proteins (81). When we aligned the TM segments from 51 different species ranging from humans to coelacanths (see Supplementary Table 1 for a complete list) a highly conserved motif was clearly identified, centered on Y145 and T150 (Figure 1B). The YxxxxT motif was invariant across these species except for a single threonine/serine difference in the alpaca sequence. Interestingly, this YxxxxT motif shares homology with the dimerization motif that was identified in the CD3ζ TM domain (Figure 1A) (60). In the CD3ζ dimer, the polar tyrosine and threonine side chains form interchain hydrogen bonds within the TM dimer interface. These data raise the possibility that the CD28 TM domain might contain a functional motif that may be involved in CD28 dimer formation.

**Figure 2:**
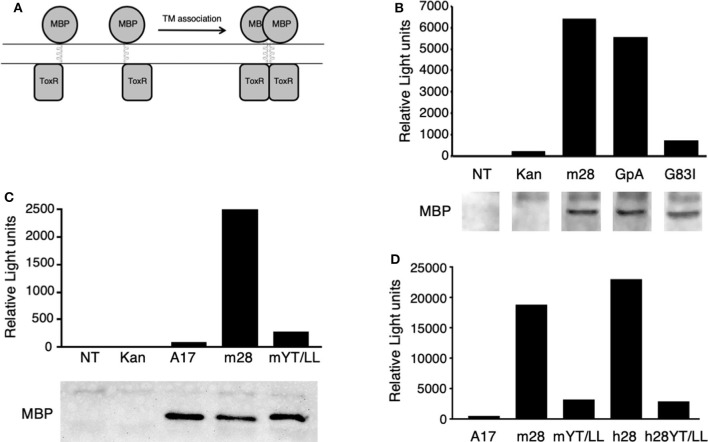
The CD28 TM can drive protein dimerization through the conserved YxxxxT motif. **(A)** The Tox-Luc system for analysis of TM domain interactions in *E. coli*. The maltose binding protein (MBP) is fused to the ToxR transcription factor through an inserted TM domain. Dimerization induced by insertion of an appropriate TM domain will activate ToxR and induce transcription of luciferase. **(B–D)** Representative experiments of luciferase activity (top) and recombinant fusion protein expression detected by MBP western blot (bottom in **B,C**) are shown. NT, untransformed *E. coli*; Kan, empty vector with no TM domain; m28, WT mouse CD28 TM; GpA, WT glycophorin A TM; G83I, mutation of GpA TM that disrupts the dimerization motif; A17, non-dimerizing polyalanine TM domain; mYT/LL, mouse CD28 TM domain containing mutations of the conserved Y145 and T150 residues to Leu; h28, WT human CD28 TM; hYT/LL, human CD28 TM containing the YT/LL mutations. The differences between m28 and GpA in **(B)** (*n* = 8) and between m28 and h28 in **(D)** (*n* = 4) are not significant. The differences between GpA and G83I in **(B)** (*n* = 7, *p* = 0.0002), m28 and mYT/LL in **(C)** (*n* = 8, *p* = 0.0015), and in **(D)** (*n* = 4, *p* = 0.0143) and between h28 and hYT/LL in **(D)** (*n* = 4, *p* = 0.0286) are significant (Mann-Whitney).

To determine whether the CD28 TM domain can mediate protein dimerization, we used the Tox-Luc system, which was derived from the Tox-CAT system originally developed by Engelman and colleagues in 1999 (77, 82) (Figure 2). The Tox-CAT system (and its derivatives with different reporters in place of CAT) has been widely used and validated to study the efficiency of TM interaction motifs in a wide variety of TM domain containing proteins (83–86). In this bacterial expression assay, inclusion of a TM dimerization motif drives the activation of the ToxR transcription factor, which in turn induces levels of luciferase expression that is proportional to the efficiency of dimerization (Figure 2A). As a positive control, we used the well-defined dimerization motif (GxxxG) present in GpA (53, 54). Insertion of the WT GpA TM domain induces luciferase expression, which is abrogated when the GpA motif is disrupted by the G83I mutation within the essential dimerization motif (Figure 2B). When the WT mouse CD28 TM domain was inserted into the ToxLuc construct, a similar level of luciferase expression was detected compared to the GpA TM domain, suggesting that the CD28 TM domain contains an efficient dimerization motif (Figure 2B).

To determine if protein dimerization mediated by the CD28 TM domain was dependent on the evolutionarily conserved YxxxxT motif, we introduced a mutated version of the mouse CD28 TM, where the Tyr and Thr residues were mutated to Leu (Y145L/T150L; YT/LL). Mutation of the conserved YxxxxT motif resulted in a dramatic loss of luciferase expression, to levels similar to a non-dimerizing control TM segment (poly-alanine) (Figure 2C). Western blot detection of the maltose binding domain (MBP) present in the fusion proteins confirmed equivalent expression of the recombinant proteins (lower panels in Figures 2B,C). The mouse CD28 TM differs from the consensus TM sequence at several locations, most notably a Ser to Gly change within the YxxxxT motif (see Figure 1 and Supplementary Table 1). To determine whether the mouse TM dimerization motif was shared with other species, we tested the human CD28 TM domain in the Tox-Luc assay. As we had seen with the mouse CD28 TM, the human CD28 TM was able to promote dimerization and this dimerization was dependent on the presence of the Tyr and Thr residues (Figure 2D). Taken together, these results suggest that the CD28 TM domain contains a potent dimerization motif that is mediated by the YxxxxT motif that is highly conserved over evolution and shares homology with the dimerization motif in CD3ζ.

To evaluate protein dimerization in T cells, we developed a flow cytometry-based assay to measure intermolecular fluorescence resonance energy transfer (FRET). We and others have previously shown that YFP/CFP FRET could be detected within the CD28 homodimer by acceptor photobleaching and fluorescence microscopy (43, 49). To detect FRET within the CD28 dimer by flow cytometry, we used cerulean fluorescent protein (CER), which has a higher quantum yield than CFP (87). In this system, FRET is measured by the sensitized emission from YFP following excitation of CER (Supplementary Figure 1). FRET was readily detected within WT CD28 dimers by flow cytometry over a 5–10-fold range of YFP and CER expression. As expected, the relative FRET efficiency increased as the level of CD28-YFP chimeras was increased, as a higher percentage of the CD28-CER chimeras would be paired with CD28-YFP and more of energy emitted from CER would be absorbed by YFP. Likewise, relative FRET efficiency decreased with increasing amount of CD28-CER (Supplementary Figures 1D–F). These data are consistent with detection of intermolecular FRET between randomly assembled dimers of CD28-YFP and CD28-CER.

**Figure 3:**
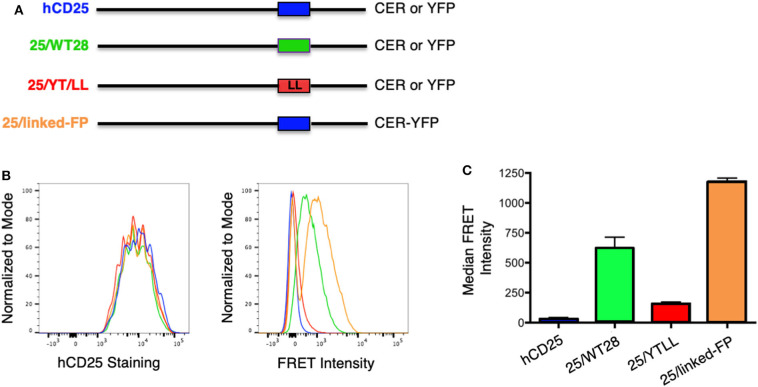
Ectopic expression of the WT CD28TM domain in monomeric hCD25 is sufficient to induce dimerization in T cell membranes. **(A)** Diagram of recombinant proteins fused to CER or YFP; hCD25, WT monomeric human CD25 (blue); 25/WT28, hCD25 containing the WT CD28 TM domain (green); 25/YT/LL, hCD25 containing the Y145L/T150L mutated CD28 TM (red); 25/linked-FP, WT hCD25 fused to both CER and YFP in tandem as a positive FRET control (orange). **(B)** T cell hybridomas were stably transduced with the hCD25 chimeric proteins and analyzed for cell surface expression of CD25 (left) and FRET intensity (right) by flow cytometry (colors as in **A**). **(C)** Median FRET intensity from independently generated triplicate cell lines with the error bars representing the standard error of the mean. The differences between all groups are statistically significant (*t*-tests, *p* < 0.01).

To determine whether the CD28 TM domain was sufficient to mediate protein dimerization in T cells, we used monomeric human CD25 as a backbone and generated YFP and CER fusions to the cytosolic tails of WT hCD25 (Figure 3A). To assure that cells expressing constructs that do and do not generate FRET are expressing similar levels of CER, we generated these cell lines by sequential transfection. We first transduced the CER constructs and bulk sorted for cells that expressed similarly broad levels of CER and then retransduced these cells with the YFP constructs. This avoids the complication where donor quenching reduces the CER signal in cells were FRET is occurring. As predicted, hCD25 is expressed at the plasma membrane as a monomer and no FRET is detected when hCD25-YFP and hCD25-CER are coexpressed at the cell surface (Figures 3B,C). A hCD25 construct in which CER and YFP were fused in tandem to the cytosolic tail was used as a positive control for FRET (Figure 3). We then generated chimeric YFP and CER fusion proteins in which the hCD25 TM domain was replaced with either the WT or YT/LL mutated CD28 TM domains (Figure 3A). These constructs, along with the hCD25 positive and negative control constructs, were sequentially transduced into CD28-negative DO11.10 T cell hybridomas to generate stable cell lines with similar levels of both FRET donor and acceptor fluorescent proteins. All of the hCD25 fusion proteins were expressed at equivalent levels on the cell surface (Figure 3B, left panel). We found that the WT CD28 TM domain was able to drive dimerization of the hCD25 construct as detected by an increase in FRET over a 5–10 fold range of YFP and CER expression (Figures 3B,C and Supplementary Figure 2). As seen for WT CD28 dimers (Supplementary Figure 1), the relative FRET efficiency increased as the level of hCD25-mCD28TM-YFP chimeras was increased and decreased as the level of hCD25-mCD28TM-CER chimeras was increased (Supplementary Figure 2), consistent with detection of intermolecular FRET between randomly assembled dimers of hCD25 mediated by inclusion of the ectopic CD28 TM domain. As seen above in the Tox-Luc assay, the ability of the CD28 TM domain to mediate dimerization was reduced when the conserved YxxxxT motif was mutated to LxxxxL (YT/LL) (Figures 3B,C and Supplementary Figure 2). These data confirm that the CD28 TM domain contains an effective dimerization motif that is sufficient to drive dimerization of monovalent hCD25 and this dimerization motif is dependent on the conserved Tyr and Thr.

**Figure 4:**
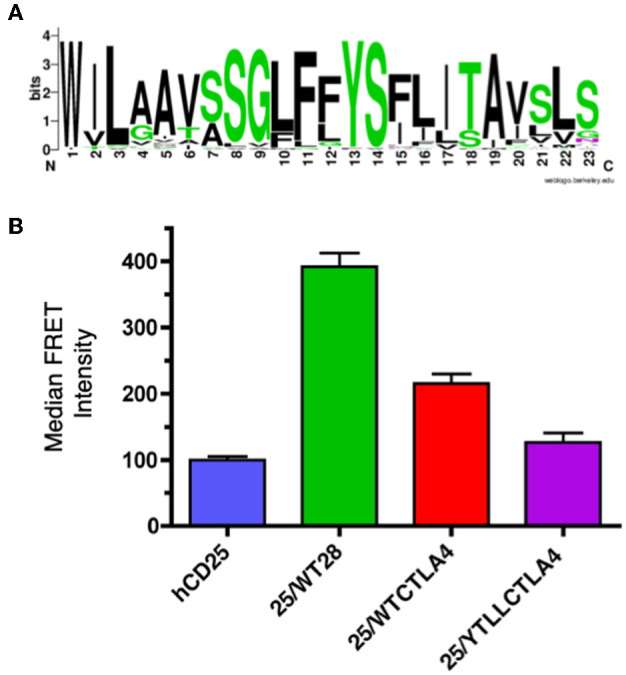
The CTLA-4 TM domain contains a conserved YxxxxT dimerization motif. **(A)** A WebLogo consensus sequence of CTLA-4 transmembrane sequences from 44 diverse species ranging from human to xenopus (see Supplementary Table 2 for a complete listing of sequences included). **(B)** The hCD25 TM domain was replaced with WT (25/WTCTLA4, red) and YT/LL mutated (25/YTLLCTLA4, purple) CTLA-4 TM domains and fused to CER or YFP as shown for the CD28 chimeras in Figure 3A. The chimeric constructs were retrovirally transduced into T cell hybridomas and analyzed for intermolecular FRET by flow cytometry. Shown are the median FRET intensity with the error bars representing the standard error of the mean. Unmodified hCD25 (blue) and hCD25 containing WT mouse CD28 TM domain (25/WT28, green) were included as negative and positive controls, respectively. The differences between all groups are statistically significant (*t*-tests, *p* < 0.01), except for hCD25 vs. 25/YTLLCTLA4 which is not statistically significant.

CTLA-4 is a close structural homolog of CD28 that binds the same ligands and antagonizes CD28 function (27, 88, 89). As with CD28, CTLA-4 is highly conserved in evolution and homologs of CTLA-4 can be found in many vertebrate species. Interestingly, analysis of the TM domain of CTLA-4 revealed the same conserved YxxxxT motif that was observed in CD28 (Figure 4A and Supplementary Table 2). However, the CTLA-4 motif is less conserved than in CD28 and most notably all the avian species examined contain a conservative threonine to serine variation. To determine if the CTLA-4 TM domain also mediated dimerization, we generated similar hCD25 chimeric constructs containing either the WT or the YT/LL mutated mouse CTLA-4 TM domain as described above for CD28. Inclusion of the CTLA-4 TM domain can induce dimerization of hCD25 as seen by an increase in FRET (Figure 4B). The level of FRET in the CTLA-4 chimeras is less than the CD28 chimeras, which suggests that the CTLA-4 TM may be less efficient at dimerization. However, it is also possible that the reduced FRET is due to a change in the orientation of the TM domains that results in an altered orientation of the YFP and CER domains fused to the cytosolic tail. Interestingly, the dimerization that is induced by the CTLA-4 TM domain is also dependent on the YxxxxT motif, as the FRET efficiency is reduced in the cells expressing the hCD25-CTLA-4-YT/LL TM chimera.

**Figure 5:**
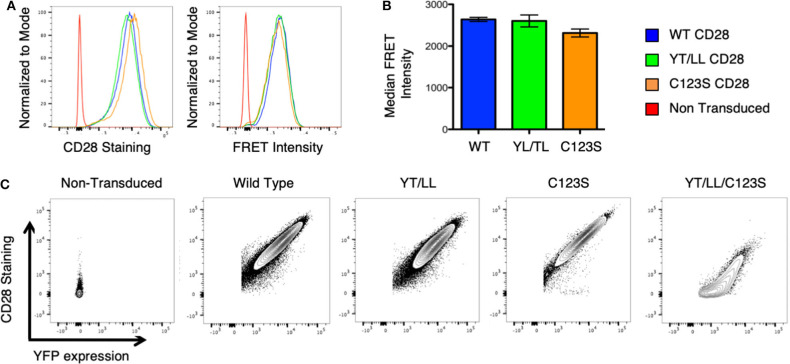
The CD28 TM domain contributes to CD28 dimerization and cell surface expression. **(A)** Retroviruses expressing WT and mutated CD28-YFP and CD28-CER constructs were transfected into CD28-negative, DO11.10 T cell hybridomas and analyzed by flow cytometry for cell surface CD28 expression (left) and FRET intensity of CD28-YFP/CD28-CER expressing cells (right). **(B)** The median FRET intensity from independently generated triplicate cell lines is shown with the error bars representing the standard error of the mean. The differences between the groups are not statistically different. **(C)** Contour plots portray the relationship between CD28 surface staining and YFP expression of CD28-YFP expressing T cell hybridomas. The relationship of YFP to CD28 expression is linear and equivalent for WT CD28 and for the YT/LL and C123S single mutants, but for the YT/LL/C123S double mutant, significant YFP expression is required before any CD28 can be detected at the cell surface, suggesting a defect in steady state surface expression of YT/LL/C123S CD28.

To assess whether the dimerization motif within the TM domain impacts CD28 assembly and/or expression, the YT/LL mutation was introduced into intact CD28 fused to CER or YFP and retrovirally transduced into CD28-deficient T cell hybridomas. Surprisingly, mutation of the TM domain did not result in a loss in CD28 dimerization as detected by FRET or a loss in CD28 cell surface expression detected by antibody staining (Figures 5A,B). CD28 has two additional dimerization motifs; one within the extracellular interaction interface and a second through an interchain disulfide bond in the stalk region (see Supplementary Figure 1A). It was possible that in the context of intact CD28 the presence of these two additional dimerization motifs could compensate for the loss of the TM domain. Therefore, we combined the YT/LL mutation with a mutation of C123 that is required for the interchain disulfide bond. As previously reported (49), mutation of C123 disrupts the formation of the interchain disulfide bond (Supplementary Figure 3), but, by itself, does not interfere with CD28 dimer formation as detected by FRET or cell surface expression (Figures 5A,B). However, disruption of both the TM dimerization motif and the interchain disulfide bond results in a dramatic defect in CD28 cell surface expression (Figure 5C). By expressing a CD28-YFP fusion protein, we can monitor both expression of total CD28 protein (detected by YFP fluorescence) and the level of CD28 at the cell surface (detected by fluorescent-Ab staining). Using this comparison, WT, YT/LL and C123S CD28 show a linear relationship between YFP and cell surface CD28, suggesting that the efficiency of dimer assembly and transport to the cell surface is similar. In contrast, this relationship is not linear in the double mutant YT/LL/C123S and a significant amount of total CD28 (as indicated by YFP levels) has to be expressed before any can be detected at the cell surface. These data suggest that the TM dimerization motif and the interchain disulfide bond both contribute to efficient stable CD28 dimer formation and the combined mutation results in a significant defect in CD28 expression at the cell surface.

**Figure 6:**
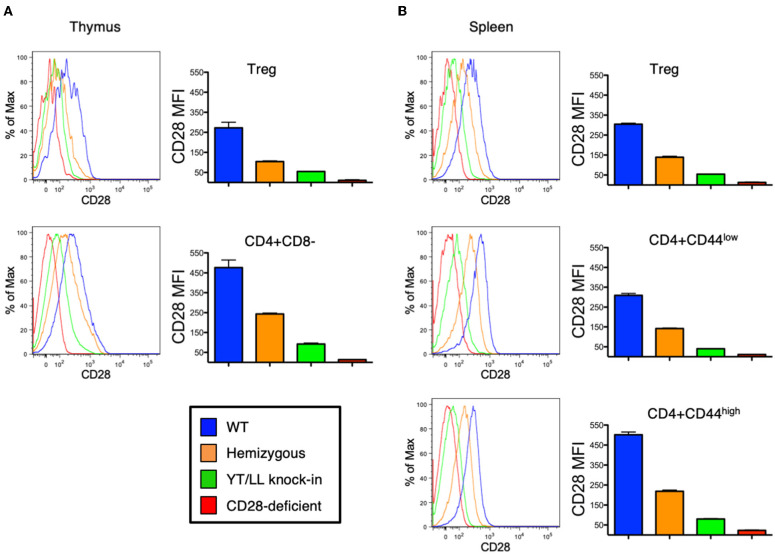
Mutation of the TM dimerization motif in mice results in a dramatic defect in CD28 expression at the cell surface. Thymic **(A)** and splenic **(B)** Treg (CD4+CD25+FOXP3+) and conventional naïve (CD44-low) and previously activated (CD44-high) CD4 T cells from WT (blue), CD28 hemizygous (orange), YT/LL knock-in (green), and CD28 knockout (red) mice were evaluated for surface expression of CD28 by flow cytometry. The distribution of CD28 staining (left) and the mean fluorescent intensities (MFI) of CD28 (right) are shown. Bar graphs depict the mean values from three individual animals, with error bars indicating the standard error of the mean. Student *t*-tests were used to evaluate statistical differences between groups. All groups showed significant differences (*p* < 0.05). Similar results were observed for 3 independent YT/LL knock-in founder lines.

As discussed above, CD28 plays a role in the development, homeostasis, activation, and function of a variety of cell types *in vivo*. Therefore, to assess whether the YT/LL mutation would affect any of these functions, we generated a genetically modified mouse, in which the YT/LL mutation was introduced into the CD28 germline using CRISPR. This allows for the appropriate level of developmental and tissue specific expression of the mutated form of CD28. We generated three independent founder lines in a homozygous C57BL/6 background. Disappointingly, all three lines demonstrated that the YT/LL mutation resulted in a dramatic decrease in CD28 cell surface expression (Figure 6). This was evident in a variety of cell types from different tissue. In addition, a similar difference in CD28 expression was detected in T cells from WT and YT/LL mice that expressed a marker of previous antigen exposure (CD44), even though these previously activated T cells expressed elevated levels of CD28 on the cell surface (Figure 6B). Unfortunately, this loss in cell surface expression precluded any functional analysis as we did not have a comparatively low expressing version of WT CD28.

**Figure 7:**
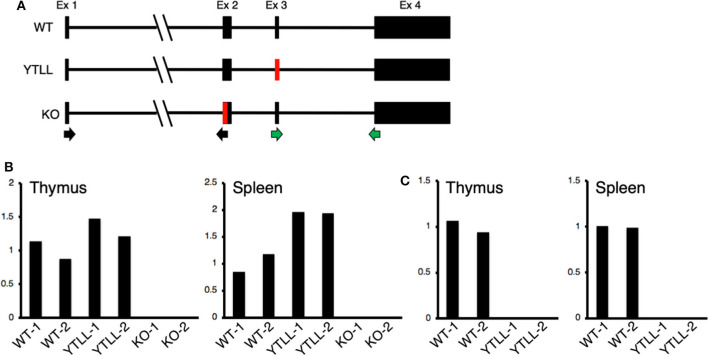
The YT/LL mutation in the endogenous CD28 locus does not result in a decrease in CD28 gene expression. **(A)** Diagram of exon/intron organization of CD28 gene. The location of the YT/LL knock-in mutation in exon 3 and the deleted region in exon 2 in the CD28 knockout mice are shown in red and the location of the exon 1/2 primer set (black arrows) and the exon 3/4 primer set (green arrows) are shown below. **(B,C)** Relative CD28 mRNA levels in thymus and spleen cells from two different WT, YT/LL, and knockout mice using the exon 1/2 primer set **(B)** or the exon 3/4 primer set **(C)**. Relative mRNA levels were normalized to GAPDH within each sample and to the mean of the two WT mRNA levels between samples.

These data suggest that under physiological conditions, the CD28 TM dimerization motif may play a critical role in the formation of stable CD28 dimers. However, in spite of introducing the YT/LL mutation into the endogenous CD28 locus, it remained possible that the loss in CD28 protein expression in the YT/LL mice was related to a defect in gene expression. To address this possibility, we compared the level of CD28 mRNA in both thymus and spleen cells derived from WT and YT/LL mice. To validate the specificity of the real-time PCR primers, we utilized two primer sets (Figure 7A). The exon 1-2 primer set amplifies mRNA from both WT and YT/LL mice, but does not amplify mRNA from the CD28 knockout mouse, which is missing a segment of exon 2 (Figure 7B). To confirm that the PCR product detected in the YT/LL cells was derived from the mutated CD28 gene, we used a second primer set that amplifies across exons 3-4. The YT/LL mutation is located at the 3′ side of exon 3 and disrupts binding of the exon 3 primer. WT mRNA, but not YT/LL mRNA, is amplified using this primer (Figure 7C). When we compared the level of CD28 mRNA in WT and YT/LL mice, we found that the levels were comparable (Figure 7B), indicating that a decrease in mRNA levels does not account for the dramatic decrease in CD28 protein expression on the cell surface. Taken together, these results suggest that under physiological conditions the YxxxxT dimerization motif within the CD28 TM domain plays a critical role in the expression of stable CD28 dimers at the cell surface.

**Figure 8:**
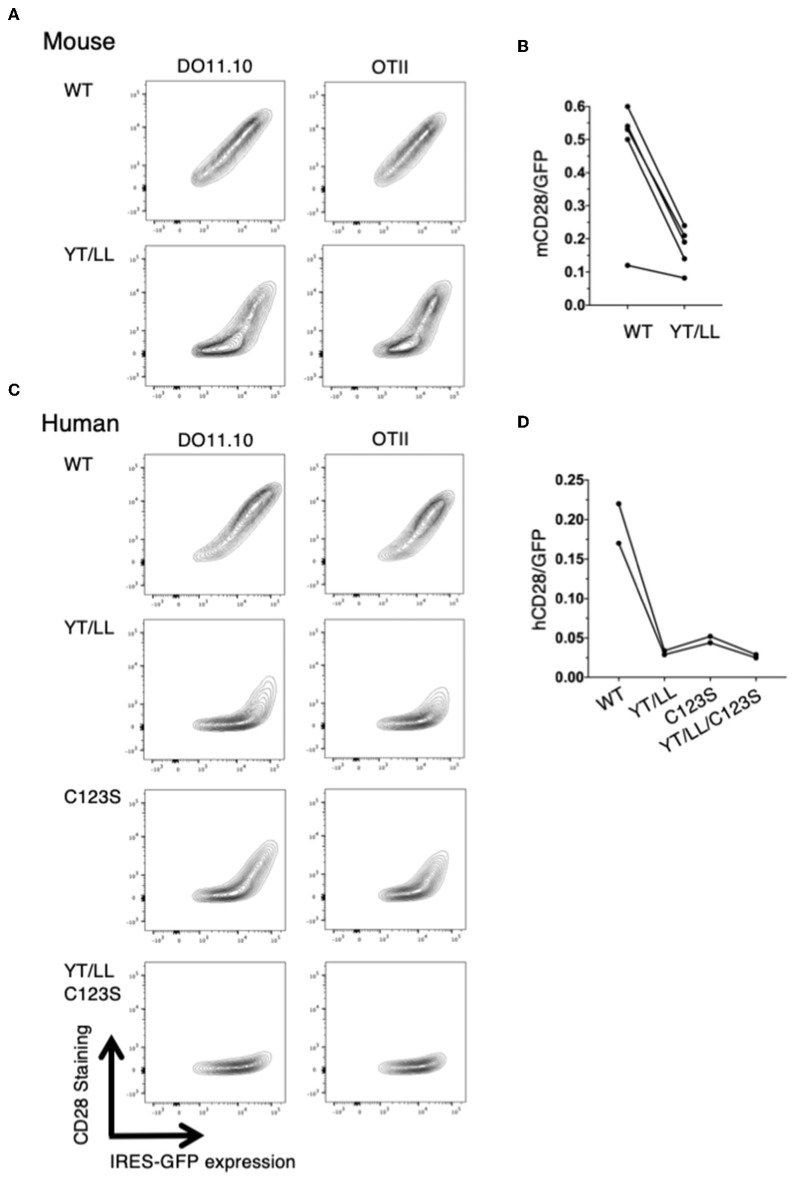
The YxxxxT motif and the interchain disulfide bond are required for stable expression CD28 on the cell surface. CD28-deficient CD4 T cells were isolated from DO11.10 or OTII TCR transgenic mice and were transduced with IRES-GFP containing retroviruses that encode WT or YT/LL mouse CD28 **(A,B)** or WT, YT/LL, C123S, or YT/LL/C123S human CD28 **(C,D)**. The cells were analyzed for cell surface expression of CD28 (y-axis) and GFP (x-axis) by flow cytometry and examples are shown **(A,C)**. The efficiency of CD28 expression at the cell surface was determined by the calculating the ratio of fluorescence intensity of cell surface staining for CD28 to the fluorescence intensity of GFP expressed in individual cells. The mean of that ratio is shown for cells expressing WT or mutated CD28 from independent experiments **(B,D)**. The difference between mouse WT and YT/LL is significant (*p* = 0.01, paired *t*-test).

The low level of CD28 cell surface expression in the YT/LL knock-in mice was surprising given our earlier result that this mutation did not affect CD28 cell surface expression (Figure 5). Because the hybridoma cells used as recipients in our transfection experiments are tumor cells, it is possible that they expressed abnormally elevated levels of ER chaperones that facilitated expression of YT/LL CD28. In addition, in these experiments, CD28 was expressed as a fusion protein to YFP or CER. Therefore, we repeated the retroviral transduction experiments using CD4 T cells isolated from untransformed CD28-deficient mice and transduced these cells with a retrovirus encoding unmodified, full length WT or YT/LL CD28 along with an IRES-GFP. We then compared the relative amount of cell surface expression of CD28 to the amount of GFP, which normalizes for the total amount of bicistronic mRNA within individual cells (Figures 8A,B). As was detected in T cells from the YT/LL knock-in mice, the efficiency of CD28 expression of YT/LL CD28 is greatly reduced compared to WT and much higher levels of overall expression (as indicated by GFP) must be achieved to accumulate CD28 at the cell surface. Similar results were obtained when we tested intact human CD28 (Figures 8C,D). When unmodified, full length WT or YT/LL human CD28 along with an IRES-GFP was transduced into untransformed CD28-deficient T cells, there was a clear defect in the level of steady state cell surface expression of human YT/LL CD28 compared to human WT CD28.

Taken together these results suggest that expression of fluorescent protein fusion proteins in tumor cells may diminish the impact of the CD28 TM dimerization motif on CD28 cell surface expression. Therefore, we reanalyzed the role of the interchain disulfide bond in intact human CD28 (Figures 8C,D). We found a clear defect in the level of steady state cell surface expression of human CD28 when the cysteine that mediates the interchain disulfide bind was mutated (C123S). The impact of the C123S mutation on human CD28 surface expression was slightly less than the YT/LL mutation and the combination resulted in nearly complete lack of stable human CD28 expression. Interestingly, the interchain disulfide is only conserved in mammals and marsupials, and is not present in birds and lower vertebrates (Supplementary Table 3). Furthermore, the length of the stalk region, that contains the interchain disulfide is highly variable in lower vertebrates. Thus, the more highly conserved TM motif supports its importance in dimer formation relative to the interchain disulfide bond.

## Discussion

In this report, we have identified a dimerization motif within the CD28 TM domain. We show that the ectopic expression of the CD28 TM domain can drive protein dimerization both in a bacterial expression system and on the cell surface of T cells. In both cases, dimerization was attenuated when the polar YxxxxT motif was mutated to hydrophobic Leu residues that are common within TM domains. A similar TM dimerization motif was first detected in CD3ζ (60) and we show that this motif is also present within CTLA-4. Interestingly, the CD28 dimerization motif may have already been inadvertently utilized to enhance the expression of chimeric antigen receptors (CAR) that contain antibody extracellular domains that detect surface antigen on tumors and cytosolic signaling domains from CD3ζ and CD28 or other costimulatory molecules (90). Thus, this motif can be added to the growing number of shared TM dimerization motifs (50, 52, 91).

It is disappointing that the requirement for the YxxxxT motif in the stable expression of CD28 at the cells surface precluded any functional analysis of the role of the CD28 TM domain in the YT/LL knock-in mice. Interestingly, the sites in the CD28 TM domain that have been identified to be positively selected for during mammalian evolution do not coincide with the YxxxxT motif (76). This raises the possibility that additional sites within the TM may play a role in CD28 signaling independent of the essential dimerization motif.

The dramatic loss in CD28 cell surface expression in T cells expressing the YT/LL or C123S mutation suggests that the TM dimerization motif and the interchain disulfide may play important roles in nucleating dimer assembly during translation in the endoplasmic reticulum and/or in the stability of CD28 at the plasma membrane. Quality control mechanisms to detect properly folded and assembled proteins are in place in both the secretory system and at the plasma membrane (92, 93). CD28 contains an additional dimer interaction site, a largely hydrophobic three-dimensional protein interface within the extracellular domain. Although protein folding can occur co-translationally, the generation of the dimerization interface within the extracellular domain that requires correct tertiary structure may be delayed compared to the availability of the interchain disulfide and TM dimerization motifs that only require secondary structure. For example, the TM domain of CD8α is both necessary and sufficient for homodimer assembly and expression even though the extracellular domain of CD8α contains an interaction motif (94). In addition to co-translation folding, oligomeric protein complexes can also assemble co-translationally (95). For homomeric complexes, co-translational assembly can occur between nascent polypeptide chains that are being synthesized by polysomes from a single mRNA (96). Thus, although the TM is near the C-terminus of CD28 and would be translated after the extracellular interaction motif, the proximity of newly synthesized proteins from polysomic mRNA might allow for TM mediated dimerization before the extracellular domain has completely folded. Alternatively, the TM interaction might be important to align the stalk region of CD28 to allow for the formation of the interchain disulfide bond and/or to allow efficient interaction between the extracellular domains. This appears to be the case for influenza neuraminidase (97, 98). Although the extracellular domain of influenza neuraminidase can assemble into a tetramer when expressed in isolation, functional assembly of neuraminidase is dependent on the TM domain when expressed as an intact protein. The formation of the tetrameric TM domain is thought to organize the orientation of the stalk regions allowing for proper interactions between the extracellular domains (98). In support of this the TM and stalk domains have co-evolved and mismatched TM and stalk domains results in a defect in neuraminidase folding and expression (97).

Alternatively, the CD28 TM may play a role in the stable expression of CD28 once it arrives at the plasma membrane. Although most of the targeting motifs for internalization and endosomal sorting have been mapped to cytosolic domains, there is evidence that TM domains can also play a role in endosomal sorting (99). For example, mutation of the TM of transferrin receptor can direct the transferrin receptor from the recycling pathway to lysosomes, resulting in a decrease in half-life (100). Likewise, mutation of a Tyr residue in the TM of Hedra virus F protein diverts the F protein from the recycling pathway to the lysosomal degradation pathway (101). In these cases, changes in endosomal sorting are associated with changes in TM domain mediated oligomerization. TM domains can also regulate interactions with ubiquitin ligases. The march family of ubiquitin ligases regulates a wide variety of immunologically relevant cell surface proteins, including for example MHC class I, MHC class II, CD86, and CD4 (102). March proteins are thought to interact with their substrates though TM domain interactions that leads to ubiquitin addition to cytosolic Lys residues, internalization from the plasma membrane, targeting into multivesicular bodies in endosomes, and eventual proteins degradation. Addition of ubiquitin to cytosolic domains can also be indirectly regulated by sequences within the TM domain, by changes in the orientation of the TM that exposes cytosolic Lys to ubiquitin ligases (103). In this regard it is interesting, that the cytosolic tail of CD28 is polybasic and is thought to interact with the negatively charged inner leaf of the plasma membrane (104, 105). Disruption of the TM domain interaction in the YT/LL mutation could alter the orientation of the cytosolic tail exposing ubiquitin ligase interaction sites.

Our data indicate that CD28 contains three distinct interaction motifs that participate in the assembly and/or stable cell surface expression of CD28 homodimers. Individual mutation of the extracellular hydrophobic interaction interface (28), the interchain disulfide bind (Figure 8) or the TM dimerization motif (Figures 6, 8) resulted in a loss of CD28 expression at the cell surface. It will be interesting to determine how these three contact site within the CD28 homodimer impact on the conformational flexibility of CD28 during inside-out and outside-in signaling during CD28-mediated costimulation of T cells.

## Data Availability Statement

All datasets generated for this study are included in the article/Supplementary Material.

## Ethics Statement

The animal study was reviewed and approved by University Committee on Animal Resources, University of Rochester Medical Center.

## Author Contributions

JM conceived of the project and drafted the manuscript. JM, SL, and MF developed experimental approaches. SL, MF, and KA contributed to revisions. All authors performed experiments, contributed, and analyzed data.

## Conflict of Interest

The authors declare that the research was conducted in the absence of any commercial or financial relationships that could be construed as a potential conflict of interest.

## Abbreviations

ER: endoplasmic reticulum
FRET: fluorescence resonance energy transfer
GpA: glycophorin A
hCD25: human CD25
TM: transmembrane
TCR: T cell receptor
YT/LL: Y145L/T150L mutation in CD28 transmembrane
WT: wild type.
