# Supplementary material for: The CD28 Transmembrane Domain Contains an Essential Dimerization Motif
Source: Front Immunol. 2020 Jul 16;11:1519. doi: 10.3389/fimmu.2020.01519 (PMC7378745; doi:10.3389/fimmu.2020.01519)
Supplement: Supplementary file 1 [file Data_Sheet_1.pdf]

Common Name	Latin name	Accession Number	Sequence
MOUSE	*Mus musculus*	AAA37396.1	WALVVVAGVLFCYGLLVTVALCVIW
HUMAN	*Homo sapiens*	NM_006139	WVLVVVGGVLACYSLLVTVAFIIFW
BABOON	*Papio anubis*	XP_009181099.1	WALVVVGGVLACYSLLVTVAFSIFW
MACAQUE	*Macaca fascicular*	XM_015432133	WALVVVGGVLACYSLLVTVAFCIFW
GREEN MONKEY	*Chlorocebus sabaeus*	XM_007965942	WALVVVGGVLACYSLLVTVAFSIFW
GIBBON	*Nomascus leucogenys*	XM_003253970	WALVVVGGVLACYSLLVTVAFSIFW
MARMOSET	*Callithrix jacchus*	XM_008999126	WALAVVGGVLASYSLLVTVALSVFW
TARSIER	*Tarsius syrichta*	XM_008074308	WALVVVGGVLVFYSLMVTVALCTFW
TREE SHREW	*Tupaia chinensis*	XM_006153077	WALTVASGILAFYGLLVTVALFAYW
RAT	*Rattus norvegicus*	NP_037253.1	WPLVVVAGVLLCYGLLVTVTLCIIW
WOODCHUCK	*Marmota monax*	AAF36501.1	WTLVVFSGVLGIYSLLSTMLLCYLW
RABBIT	*Oryctolagus cuniculus*	XP_017198104.1	WVLVVVGAVLAFYSMLVTVALFSCW
DOG	*Canis familiaris*	NP_001003087.2	WALVVVGAVLVFYSLLVTVALCAYW
CAT	*Felis catus*	NP_001009844.1	WALVVVGGILGFYSLLATVALGACW
SIBERIAN TIGER	*Panthera tigris altaica*	XP_007090030.2	WALVVVGGILGFYSLLATLALCACW
FERRET	*Mustela putorius*	XM_013062342	WALVVVCGVLVFYSFLVTVALCACW
GIANT PANDA	*Ailuropoda melanoleuca*	XM_002919949	WALVVVGGVLVFYSLLVTVALCACW
ALPACA	*Vicugna pacos*	XP_006205268.1	WALVVVASILAFYSLLASVALCNCW
SHEEP	*Ovis aries*	XM_012111193	WALVVVNGVLVFYSLLVTVALCNCW
BROWN FLYING FOX	*Pteropus alecto*	XP_006921437.1	WALVVVGGVLAFYSLLVTMALCICW
BROWN BAT	*Myotis lucifugus*	XM_014461937	WALVVVVGVLALYSLLVTVALFTYW
HORSE	*Equus caballus*	NM_001100179	WALAVTGGVLAFYSLLVTVALCTCW
WHITE RHINOCEROUS	*Ceratotherium simum*	XP_004426878.1	WALVVV-GVLAFYSLLVTVALCTCW
AFRICAN ELEPHANT	*Loxodonta africana*	XM_003405929	WALVVVNGVLAFYSLVITVALCICW
ARMADILLO	*Dasypus novemcinctus*	XM_012523351	WLL-ALLGLLGLYSLLATVCLFIYW
KILLER WHALE	*Orcinus orca*	XP_004262874	WALVVVNGVLAFYSLLATVALSNCW
BOTTLENOSE DOLPHIN	*Tursiops truncatus*	XP_004315141	WALVVVNGVLAFYSLLATVALSNCW
GREY-TAILED OPOSSUM	*Monodelphis domestica*	XP_007501953.1	WPVVAALCVFAFYSMLITVAFCNCW
TASMANIAN DEVIL	*Sarcophilus harrisii*	XM_003766002	WAVVAALCVLASYSMLITVAFFNCW
KOALA	*Phascolarctos cinereus*	XP_020828504.1	WAVTAALCILAFYSLLMTVAFLNCW
STARLING	*Sturnus vulgaris*	XM_014880339	WILGTVTGILSLYSMLVTAVCINYW
GREAT TIT	*Parus major*	XP_015490581.1	WILATVTGILALYSMLITAVFINYW
CHICKEN	*Gallus gallus*	NM_205311	WVMVAVTGLLGFYSMLITAVFIIY
TURKEY	*Meleagris gallopavo*	CAP04926.1	WVMVALTGLLGFYSMLVTAVFIIY
DALMATIAN PELICAN	*Pelecanus crispus*	XP_009488081.1	WTTVAVTGVLAFYSMLITAVFINYW
BALD EAGLE	*Haliaeetus leucocephalus*	XP_010563686.1	WIMVAVTGVLAFYSMLITAVFINYW
RUFF	*Calidris pugnax*	XM_014954243	WIMVAVTGVLAFYSMLITAVFINYW
ALLIGATOR	*Alligator mississippiensis*	XM_006261310	WLMAAAIGVFAIYSIIITVACIHCS
SALTWATER CROCODILE	*Crocodylus porosus*	XP_019410247	WLMAAAIGVFAIYSIIITVACIHCS
GREEN SEA TURTLE	*Chelonia mydas*	XP_007071905.1	WALVVTLGFLAFYSVLITAAFVICW
SOFTSHELL TURTLE	*Pelodiscus sinensis*	XM_014577908	WPIVVTLGVLAFYGVLVTAAFVICW
CLAWED FROG	*Xenopus tropicalis*	XM_012969996	WPLLVAIGVMAAYSLTITAAFFYIL
BURMESE PYTHON	*Python bivittatus*	XP_025031234.1	VATGIPLGFFVLYSVIITAAVCYCW
PIT VIPER	*Protobothrops mucrosquamatus*	XM_015819766.1	MPMAIPLGFFVFYSMIITAAIYYCW
ANOLE	*Anolis carolinensis*	XM_008114951	MLMIAAIIIFIVYSVTVTAACYYCW
BEARDED DRAGON	*Pogona vitticeps*	XP_020670148.1	TWMTGTIGGLVLYGIAITAAFCYCW
RAINBOW TROUT	*Oncorhynchus mykiss*	NP_001118004.1	WAWMLGFWVTTIYGLVVTVFAFVIW
ATLANTIC SALMON	*Salmo salar*	ACI33726.1	WAWVLGFWVTTIYGLVVTVIAFVIW
PUFFER FISH	*Takifugu rubripes*	NP_001267008.1	GWMWIFALVIGSYSVTVTIGAIIIW
KILLIFISH	*Fundulus heteroclitus*	XP_012712711.2	WIWIMVVALLGTYSLAVTIAAFIIW
COELACANTH	*Latimeria chalumnae*	XM_014490127	WLPICLTAFFLVYSMTITIMYCNLQ

Supplemental Table 1. The YxxxxT motif in the CD28 TM domain is evolutionarily conserved. The TM domain sequences of CD28 homologs from 51 different species are shown for mammals, marsupials, birds, reptiles, and fish. The conserved YxxxxT motif is highlighted in green and the single T/S variant in the alpaca is highlight in yellow.

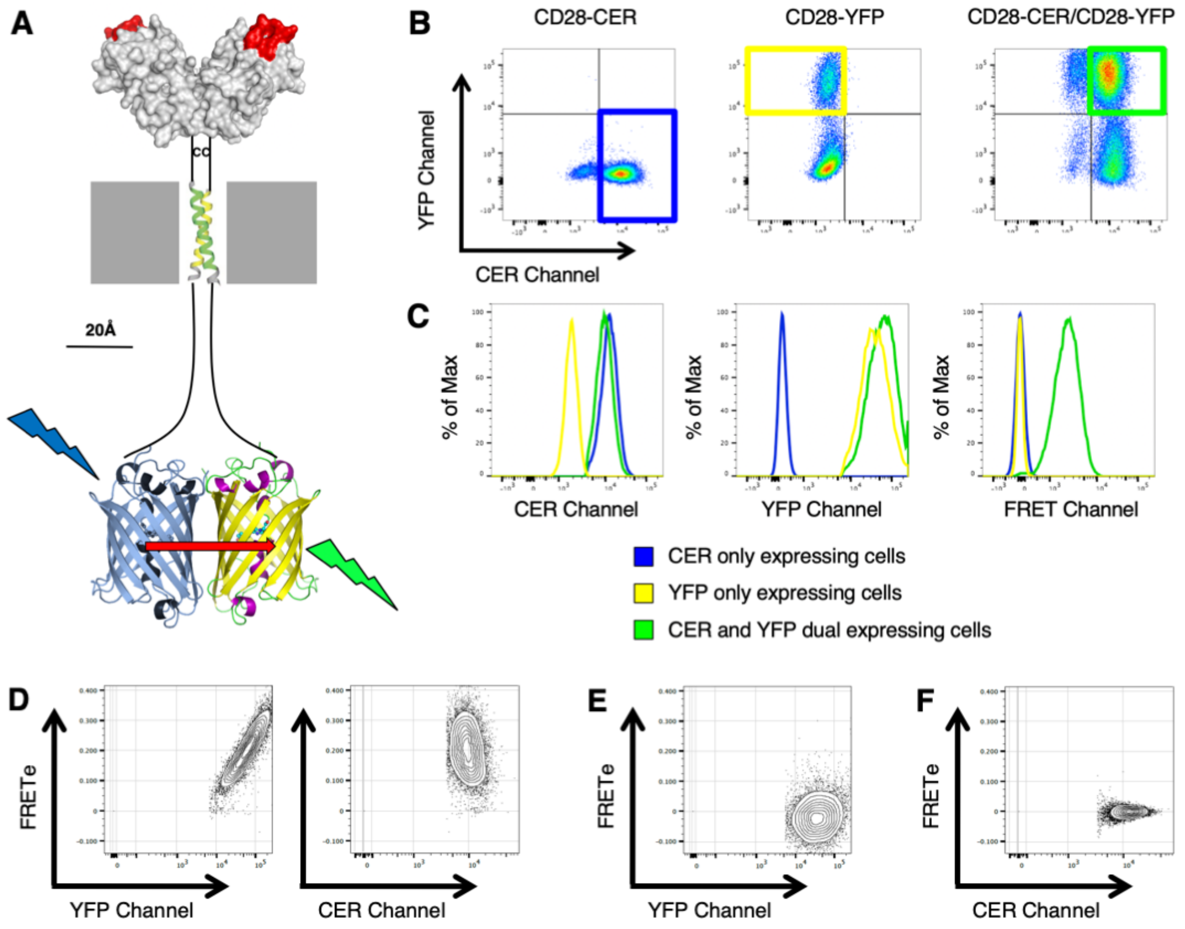

Supplemental Figure 1. Analysis of intermolecular FRET by flow cytometry. (A) Diagram of the CD28 dimer fused at the C-terminus to CER and YFP. When the tails of a CER-YFP containing dimer are within 100Å of each other, CER acts as a donor and YFP as an acceptor for FRET when excited by a 405nm laser and sensitized FRET emission is detected at 550nm. **(B)** CD28 deficient DO11.10 CD4 T cell hybridoma cells were retrovirally transduced with either CD28-CER, CD28-YFP, or both and were gated on expressing cells. Left, cell expressing only CD28-CER (blue box); middle, cell expressing only CD28-YFP (yellow box); right, cells expressing both CD28-CER and CD28-YFP (green box). **(C)** Fluorescent profiles of CD28-CER, CD28-YFP, or dual expressing cells as gated in **(B)** for the CER, YFP, or FRET channels. (**D-F**) Relative FRET efficiency (FRETe) was calculated and plotted by YFP or CER for dual expressing cells (**D**) or by YFP for YFP only cells (**E**) or CER for CER only cells (**F**).

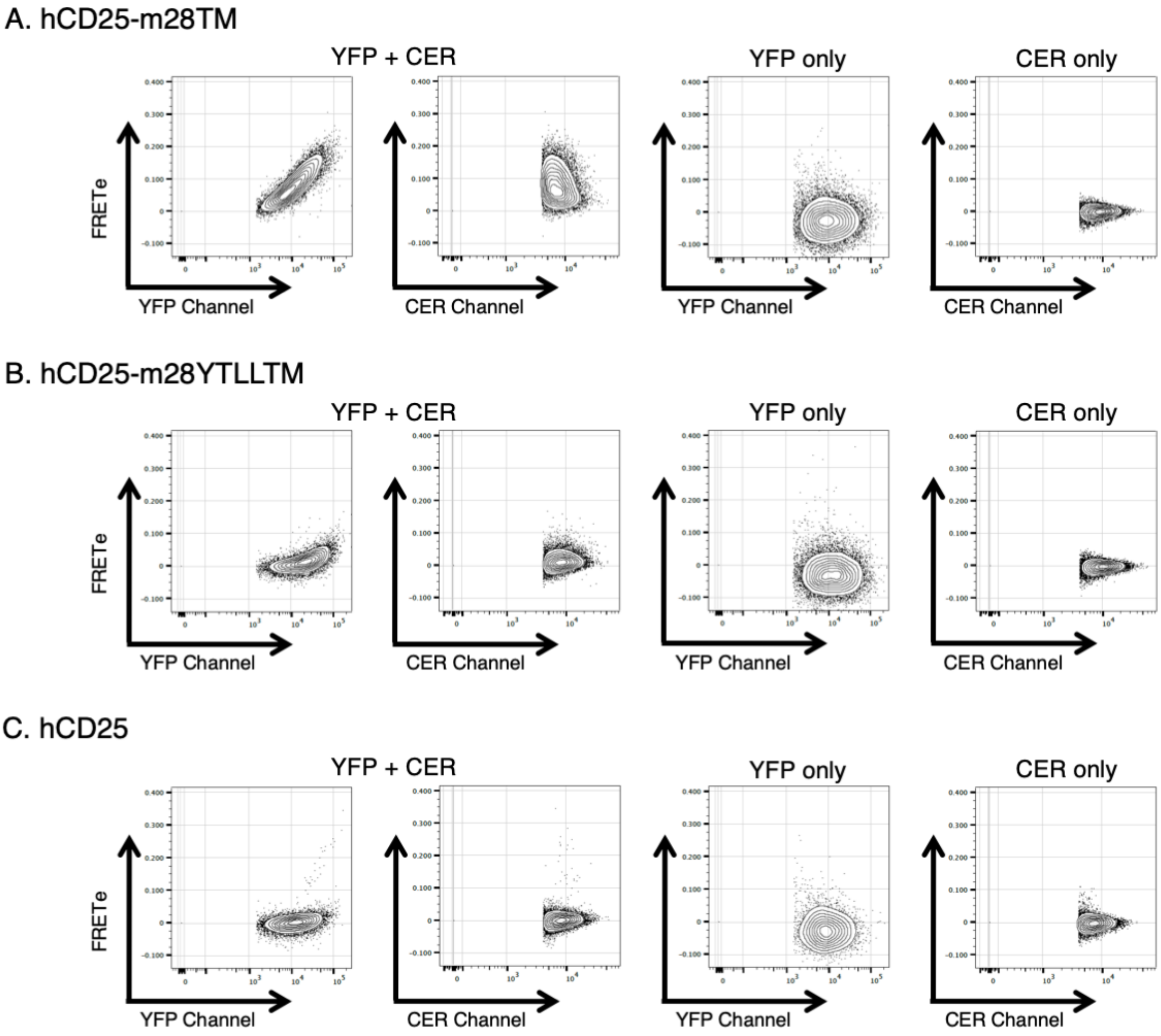

Supplemental Figure 2. FRET analysis of hCD25 chimeras expressed in T cells. Relative FRET efficiency (FRETe) was calculated for T cells expressing YFP and CER hCD25 chimeras containing the WT mouse CD28 TM domain (**A**) hCD25 chimeras containing the mouse CD28 YT/LL mutated TM domain (**B**) or unmodified hCD25 (**C**). Relative FRETe was plotted by YFP or CER for dual expressing cells (YFP + CER) or by YFP for YFP only cells or CER for CER only cells.

Common name	Latin name	Accession Number	Sequence
MOUSE	*Mus musculus*	NM_009843	WILVAVSLGLFFYSFLVTAVSLS
HUMAN	*Homo sapians*	NP_005205.2	WILAAVSSGLFFYSFLLTAVSLS
BABOON	*Papio anubis*	NP_001106104.1	WILAAVSSGLFFYSFLLTAVSLS
MACAQUE	*Macaca fascicularis*	XP_005574071.1	WILAAVSSGLFFYSFLLTAVSLS
GREEN MONKEY	*Chlorocebus sabaeus*	XP_007964134.1	WILAAVSSGLFFYSFLLTAVSLS
GIBBON	*Nomascus leucogenys*	XP_003254019.1	WILAAVSSGLFFYSFLLTAVSLS
MARMOSET	*Callithrix jacchus*	XP_002749715.1	WILAAVSSGLFFYSFLLTAVSLS
TARSIER	*Tarsius syrichta*	XP_008072501.1	WTLAAVSSGLFFYSFFLTAVVLS
TREE SHREW	*Tupaia chinensis*	XP_006153140.1	WILAAVSSGLFFYSFLITAVSLS
RAT	*Rattus norvegicus*	NM_031674.1	WILAAVSSGLFFYSFLVTAVSLN
MARMOT	*Marmota mormota*	XP_015336776.1	WILAAVSSGLFFYSFLVTAVSLS
RABBIT	*Oryctolagus cuniculus*	NP_001076154.1	WILAAISSGLFFYSFLITAVSLS
DOG	*Canis familiaris*	NP_001003106.1	WILAAVSSGLFFYSFLITAVSLS
CAT	*Felis catus*	NM_001009236	WILAAVSSGLFFYSFLITAVSLS
SIBERIAN TIGER	*Panthera tigris altaica*	XP_006921439.1	WILAAVSSGLFFYSFLITAVSLS
FERRET	*Mustela putorius*	XP_004764261.1	WILAAVSSGLFFYSFLITAISLS
GIANT PANDA	*Ailuropoda melanoleuca*	XP_002919994.1	WTLAAVSSGLFFYSFLITAVSLS
ALPACA	*Vicugna pacos*alpaca	XP_006205269.1	WVLASISSGLFFYSFLITAVSLS
SHEEP	*Ovis aries*	NM_001009214.1	WILAAVSSGLFFYSFLITAVSLS
BROWN FLYING FOX	*Pteropus alecto*	XP_006921439.1	WILAAVSSGLFFYSFLITAVSLS
BROWN BAT	*Myotis lucifugus*	XP_006082485.1	WILAAVSSGLFFYSFLITAVSLS
HORSE	*Equus caballus*	XP_023478240.1	WILAAVSSGLFFYSFLITAVSLS
AFRICAN ELEPHANT	*Loxodonta africana*	XP_003406166.1	WILAAVSSGLFFYSFLITAVSLS
ARMADILLO	*Dasypus novemcinctus*	XP_004453677.1	WILAAVSSGLFFYSFFITAISLS
KILLER WHALE	*Orcinus orca*	XP_004262872.1	WILAAVSSGLFFYSFLITAVSLS
BOTTLENOSE DOLPHIN	*Tursiops truncates*	XP_019794455.1	WILAAVSSGLFFYSFLITAVSLS
OPOSSUM	*Monodelphis domestica*	XM_007501888.2	WILAIVSSGLFFYSLLITAVSLN
TASMANIAN DEVIL	*Sarcophilus harrisii*	XM_003766000.2	WILAIVSSALFFYSFLITAVSLN
PLATYPUS	*Ornithorhynchus anatinus*	XM_001514865.2	WILAAVSSGLFIYSFLITMVALS
STARLING	*Sturnus vulgaris*	XP_014735824.1	WVLGATASGFFLYSIIISAILVG
GREAT TIT	*Parus major*	XP_015490579.1	WVLGATASGFFLYSIIISAILVG
CHICKEN	*Gallus gallus*	NM_001040091.1	WVLGATASGFFLYSIIISAIVVG
TURKEY	*Meleagris gallopavo*	XM_019617059.1	WVLGATASGFFLYSIIISAIVVS
DALMATIAN PELICAN	*Pelecanus crispus*	XP_009478785.1	WVLGATASGFFLYSIIISAILVG
BALD EAGLE	*Haliaeetus leucocephalus*	XP_010563675.1	WVLGATASGFFLYSIIISAILVH
RUFF	*Calidris pugnax*	XP_014809732	WVLGATASGFFLYSIVISAILVG
PIT VIPER	*Protobothrops mucrosquamatus*	XM_015818576.1	WILVPVVSGLLGYSILITIFIMV
ANOLE	*Anolis carolinensis*	XM_008125603.2	WISVMIASGLLGYSILITIYM
ALLIGATOR	*Alligator mississippiensis*	XM_006267243.3	WILGAAASGLFLYSIIISAFLLS
SALTWATER CROCODILE	*Crocodylus porous*	XP_019409174.1	WILGAAASGLFLYSIIISAFLLS
GREEN SEA TURTLE	*Chelonia mydas*	XM_007071927.1	WILGAAASGLFIYSILITACVLS
BURMESE PYTHON	*Python bivittatus*	XP_007442144.2	WILILVASGLLGYSILITIFIM
BEARDED DRAGON	*Pogona vitticeps*	XP_020636419.1	IIVTVVAVVFGLFVNSILITVYIT
CLAWED FROG	*Xenopus tropicalis*	XM_018097345.1	ILLVVCLVMFLYSMFITAVLLCG

Supplemental Table 2. CTLA4 also contains an evolutionarily conserved YxxxxT motif. The TM domain sequences of CD28 homologs from 44 different species are shown for mammals, marsupials, birds, and reptiles. The conserved YxxxxT motif is highlighted in green, conservative aa variants (T/S and Y/F) are highlight in yellow, and the single nonconservative aa variant in the bearded dragon (T/L) is highlighted in red.

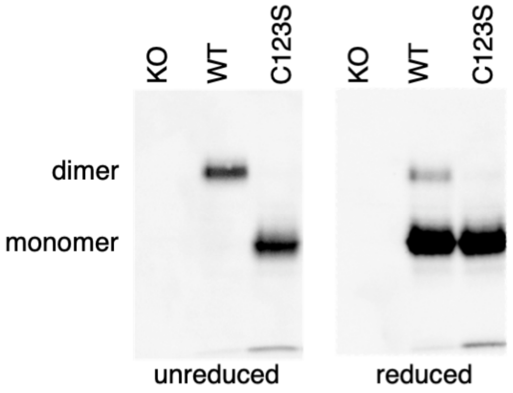

Supplemental Figure 3. C123S disrupts CD28 disulfide bond formation. Retroviruses expressing WT and C123S CD28 containing a C-terminal HA tag were transfected into CD28-deficient, DO11.10 T cells (KO) and lysates were analyzed by nonreducing and reducing SDS-PAGE and western blotting with anti-HA Ab.
